# The impact of the health care workforce on under-five mortality in rural China

**DOI:** 10.1186/s12960-019-0357-5

**Published:** 2019-03-18

**Authors:** Siyuan Liang, James Macinko, Dahai Yue, Qingyue Meng

**Affiliations:** 1 School of Public Health, Peking University, Beijing, 100191 China; 2 China Center for Health Development Studies, Peking University, Beijing, 100191 China; 3 Fielding School of Public Health, University of California, Los Angeles, Los Angeles, CA 90095 United States of America

**Keywords:** Under-five mortality, China, Health system, Human resources for health

## Abstract

**Background:**

Previous studies have focused on the relationship between increases in the health care workforce and child health outcomes, but little is known about how this relationship differs in contexts where economic growth differs by initial level and pace. This study evaluates the association between increased health professionals and the under-five mortality rate (U5MR) in rural Chinese counties from 2008 to 2014 and examines whether this relationship differs among counties with different patterns of economic growth over this period.

**Methods:**

We estimated fixed effects models with rural counties as the unit of analysis to evaluate the association between health professional density and U5MR. Covariates included county-level gross domestic product (GDP) per capita, female illiteracy rate, value of medical equipment per bed, and province-level health expenditures (measured as a proportion of provincial GDP). To explore modification effects, we assessed interactions between health professionals and county types defined by county poverty status and county-level trajectories of growth in GDP per capita. U5MR data have been adjusted for county-level underreporting, and all other data were obtained from administrative and official sources.

**Results:**

The U5MR dropped by 36.19% during the study period. One additional health professional per 1000 population was associated with a 2.6% reduction in U5MR, after controlling for other covariates. County poverty status and GDP trajectories moderated this relationship: the U5MR reductions attributed to a one-unit increase in health professionals were 6.8% among poor counties, but only 1.1% among non-poor ones. These reductions were, respectively, 6.7%, 0.7%, and 4.3% in counties with initially low GDP that slowly increased, medium-level GDP that rose at a moderate pace, and high GDP that rose rapidly.

**Conclusions:**

This study demonstrates that increased health professionals were associated with reductions in U5MR. The largest association was seen in poor counties and those with low and slowly increasing GDP per capita, which justifies further expansion of the health care workforce in these areas. This study could be instructive for other developing countries to achieve Sustainable Development Goal 3 by helping them identify where additional health professionals would make the greatest contribution.

**Electronic supplementary material:**

The online version of this article (10.1186/s12960-019-0357-5) contains supplementary material, which is available to authorized users.

## Background

Child mortality reduction was a global target of the Millennium Development Goals (MDGs) and is included in the current Sustainable Development Goals (SDGs). China is one of the few countries that has already achieved child health goals for both MDG4 (reduce under-five mortality by two thirds between 1990 and 2015) and SDG3 (reduce under-five mortality to 25/1000 live births or less by 2030). This accomplishment merits further evaluation, both to understand the Chinese experience and to provide lessons for other developing countries undergoing health reform alongside rapid social and economic development [1].

The determinants of child mortality have been extensively explored. Previous studies have established evidence-based child survival frameworks that identify three distinct levels of determinants [2–5]. Distal determinants (governance, conflict, environment) influence intermediate determinants (individual-, household-, and community-socioeconomic factors) which function via the proximate determinants (maternal and child characteristics, injury/personal illness control) to directly affect the risk of child morbidity and mortality [2–5].

The majority of studies on drivers of child mortality reductions showed that the most important determinants were health system factors (health financing, health workforce, vaccination, and other treatments), maternal education, household wealth level, and access to clean water and sanitation [4, 6–10]. Most child survival frameworks identify the health system as critical in reducing child mortality. For example, one study of 146 low- and middle-income countries found that improvements within the health sector (e.g., health service coverage, immunizations) accounted for approximately 50% of maternal and child mortality reductions from 1990 to 2010 [7]. According to Mosley and Chen, health systems contribute to higher child survival rates through institutionalized actions (e.g., quarantine, immunizations), cost subsidies which change the relative prices of health care services, public information/education/motivation, and technology (e.g., oral rehydration salts) [3].

Within health systems, the health care workforce is a core component, since every health system function is either undertaken or mediated by health workers [11–14]. They prevent disease through disease-control measures such as vaccination programs for children [8, 15], help mothers develop healthy behaviors like early initial breastfeeding to boost children’s nutrition and immune system [2, 4, 8, 16], and provide curative health care services which could directly save children from life-threatening diseases [2, 17–19]. As far as child health is concerned, the health care workforce is required for the provision of medication, prenatal care, and pediatric services [17]. Extensive evidence has confirmed the contribution of human resources for health to lower child mortality. Longitudinal studies from the United States of America [20], Japan [21], Brazil [22], Vietnam [14], Mozambique [23], and Lesotho [24] reported that the density of health care workforce was negatively associated with the infant mortality rate (IMR) and under-five mortality rate (U5MR). This conclusion has also been reached in several cross-country studies with both longitudinal [17, 25, 26] and cross-sectional designs [11, 12, 18, 27].

Most studies of China have assessed the role of distal and intermediate determinants, focusing on economic, social, political, health system and policy, specific health interventions, family planning, culture/custom, and environmental factors [2, 9, 10, 16, 28, 29]. However, there have been limited evaluations regarding health workers and child mortality despite the large-scale health reform in China beginning in 2009. Anand et al. illustrated that a 1% increase in the density of health professionals and technical personnel led to a 0.133% decrease in IMR at a county level in 2000 [16]. Guo et al. reported that the number of health professionals per capita was negatively associated with IMR among poor rural counties of five provinces in 1997 [29]. A longitudinal study based on 116 maternal and child mortality surveillance sites between 1996 and 2002 found that, except coastal cities, health resources were the main factor that limited further decline of IMR, but the study did not quantify the direct impact of the health care workforce on IMR [28]. At a province level, Feng et al. explored the determinants of U5MR over the period 1990–2006 and revealed that the health system and policy factor (including density of health workers and density of doctors) was positively associated with U5MR while the health program and intervention factor (including postpartum and neonatal visit) had a negative association [2]. In terms of studying a local context, studies based in China have so far considered mostly unchanging characteristics such as the geographic region (eastern, central, and western China) [30], poverty status (poor and non-poor) [29], and urban or rural designation [28].

Although accumulating studies have examined the relationship between the health care workforce and U5MR in low-income and middle-income countries, the literature still has several gaps. For instance, only a few studies used longitudinal data below the national or state/province level, including studies using data from China [2, 16, 29]. Furthermore, some studies analyzed the association of the health care workforce along with health care service provision, which could lead to over-control since the relationship between workforce and U5MR is mediated by services, thus limiting the ability of these studies to estimate the total effects of the health care workforce on U5MR [2, 13]. The consensus among existing literatures is that the magnitude of this association varies in different contexts. These contexts most often refer to static characteristics or values, such as country type and geographic region [30, 31]. Nonetheless, many of these characteristics cannot easily be changed and the importance of such fixed characteristics may quickly fade in the context of the rapid economic transitions seen in many countries today. Finally, previous studies using cross-sectional data focused on the absolute level of a country’s economy, while potentially overlooking another potential distal factor: it is not only the overall size of the country’s economy (most often measured by gross domestic product, or GDP), but the pace of economic growth over time that may also affect U5MR declines. Despite economic changes that have been witnessed by almost every country, to date, no study (including those in China) has examined the association between the health care workforce and U5MR in terms of the trajectory of economic change.

China has made several efforts to strengthen its health system since the health reform in 2009, including actions to enhance the health care workforce, which provides an appealing context for this study. Before 2009, China faced a host of human resources for health issues related to a shortage of qualified staff, low compensation, and high workload, especially in primary health care institutions [32]. The 2009 reform increased investment, such as an additional US$ 127 billion to develop infrastructure and expand the health care workforce in thousands of health facilities at county, town, and village levels in rural areas, and in community health centers and stations in cities [33, 34]. The reform also helped establish a more efficient and standardized operating mechanism for health institutions, such as reasonable headcount allocation and scientific performance appraisal, which was intended to increase the motivation and retention of health professionals [33]. It is noteworthy that economically disadvantaged areas received more financial and policy support than previously [33, 35].

This article evaluates the association between increases in the number of health professionals after health reform in 2009 and U5MR in rural China. It also explores whether this association differs given the diversity of counties and their pace of economic growth over this period. We hypothesized that increased number of health professionals in a given county would be associated with decreased U5MR and that this association would be larger in economically disadvantaged counties and in counties with low initial GDP that rose more slowly over time than in other counties. Results of this study could inform future health human resources planning to improve child health outcomes in other developing countries.

## Methods

This study employed panel data with the county (one of the smallest administrative units in China) as the unit of analysis to evaluate the association between health professional density and U5MR in rural China.

### Data sources

We constructed a county-level panel dataset based on routinely reported data from the National Health and Family Planning Commission of the People’s Republic of China, National Bureau of Statistics of the People’s Republic of China, Provincial Bureau of Statistics, and National Population Census Database, and province-level data from the China Health and Family Planning Statistical Yearbook [36]. Data were collected by the county Bureau of Statistics using consistent measurements across years and counties, which are then reported to municipal Bureau of Statistics and then to provincial Bureau of Statistics. The list of counties’ poverty status was obtained from The State Council Leading Group Office of Poverty Alleviation and Development [37]. Counties were officially designated as poor according to a set standard varied by province, such as net income per capita, GDP per capita, and fiscal revenue per capita [38, 39].

The data covered the period before (2008) and after (2010, 2012, and 2014) China’s health reform. The original numbers of rural counties were 2035 (2008), 2035 (2010), 2034 (2012), and 2033 (2014), totaling 8137 county-year units. After imputation (75.21% of the observations were imputed for the female illiteracy rate), excluding extreme outliers (176 observations or 2.16% had zero U5MR, zero hospital-based health professionals, missing values for hospital-based health professionals, or negative female illiteracy rates), excluding counties with only 1 year of data (5 observations or 0.06%), and excluding missing values (303 observations or 3.81%), the final number of observations was 7653 (1956 in 2008, 1909 in 2010, 1859 in 2012, and 1929 in 2014). Though the panel was unbalanced, 92.13% (1802) of counties had data covering all 4 years. Altogether, the final included counties had an average of 894 million people. See Additional file 1: Table A1 of Appendix 1 for further information on missing data.

### Conceptual framework and measurements

**Fig. 1:**
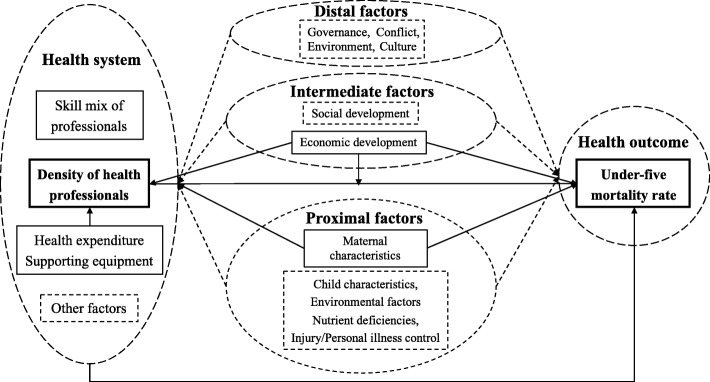
Conceptual framework illustrating hypothesized relationships between the density of health professionals and under-five mortality

Our conceptual framework is based on existing theories and literature and uses a directed acyclic graph (DAG) (Fig. 1) to illustrate hypothesized causal relationships between the density of health professionals and under-five mortality and to guide model specifications [2–5].

Our conceptual framework includes five parts in the dashed oval, as follows: The health system includes health expenditures, health professionals, equipment, and other components such as governance and leadership. Health professionals are defined by their density and skill mix. The main health outcome is U5MR. Proximal factors are grouped into five categories: maternal characteristics (age, education, parity, birth interval), child characteristics (gender, birth order, size at birth), environmental factors (air quality, access to improved water/sanitation facilities), nutrient deficiencies, and injury/personal illness control (access to preventive/curative services) [2–5]. Intermediate factors include economic development and social development [2–5]. Distal factors include governance (state regime, political stability, policy, income inequality), conflict (terrorism incidents, refugee populations), environment (natural disasters, urbanization), and culture (traditions, norms) [2–5].

Distal, intermediate, and proximal factors could affect both the health system and health outcomes, but we are unable to include all potential confounders due to constraints imposed by the availability of county-level data. Observed variables are shown in solid boxes, while unobserved variables are in dashed boxes. The effects of observed variables are represented using solid arrows while those of unobserved variables are using dashed arrows.

This study focused on the impact of health professional density on under-five mortality, so we tried to control all the measurable confounders of this relationship, both inside and outside the health system.

Under-five mortality was measured as the number of deaths among children under 5 years of age per 1000 live births. In this study, we used estimates of U5MR which had been adjusted for underreporting utilizing a validated small area mortality estimation model, spatiotemporal smoothing, and Gaussian process regression [40].

The density of health professionals was measured as the number of health professionals per 1000 population in each county. These health professionals include physicians, assistant physicians,^1^ nurses, public health physicians, pharmacists, and laboratory health workers who were registered in their affiliated health institutions [36]. Data on health professionals were obtained from the National Health and Family Planning Commission.

Economic development was controlled since counties with more prosperous economies could attract more health professionals and provide more nutritious food for children, and it could also modify the relationship between health professional density and U5MR [24, 41, 42]. Economic development was measured as GDP per capita, and poverty type was treated as a moderator. Data were obtained from the National Bureau of Statistics.

Some maternal characteristics (female illiteracy rate) were also controlled. Female illiteracy rate has been recognized as an indicator for social development [14, 43], since women with lower education would have lower child health problem recognition and higher barriers to utilize health care services (note that in the framework the arrow is on health system rather than the density of health professionals) [44]. They are also less likely to develop healthy behaviors and face barriers to adequate living conditions such as sanitation [14, 42, 43, 45]. The female illiteracy rate was measured as the number of illiterate females divided by the total female population over 15 years old. Missing data on the female illiteracy rate were imputed using a non-linear interpolation and extrapolation approach that modeled within-county changes in relation to existing values at the county level and contemporaneous values at the province level (see Additional file 1: Appendix 2) [46]. Data were obtained from county-level census data and China Health and Family Planning Statistical Yearbook [36].

The other two main confounders within the health system were health expenditures and supporting equipment. Health expenditures should affect the density of health professionals (due to the headcount quota system in China) and U5MR. Since there were no statistics on health expenditures at the county level and the health expenditure was highly correlated with GDP, the contemporaneous province-level ratio of health expenditure to GDP was chosen as a proxy for county-level health expenditures. We divided all counties into two groups by the national ratio in each year; counties below the national ratio were classified as low while others were classified as high. Health expenditure data were from the China Health and Family Planning Statistical Yearbook [36].

Supporting equipment should affect the provision of services for children and U5MR. In terms of curative services, evidence has shown that emergency obstetric care and newborn intensive care improve the child survival rate [8, 19, 31]. Therefore, we adopted the value of medical equipment per bed (equipment whose value is above 10 000 Chinese Yuan) as a proxy for the health system’s capability to address these conditions. Data were obtained from the National Health and Family Planning Commission.

Some distal factors are considered as time-invariant such as local cultural and environmental factors (terrain), so they were controlled for by including county fixed effects. Year fixed effects were incorporated to account for secular trends.

**Fig. 2:**
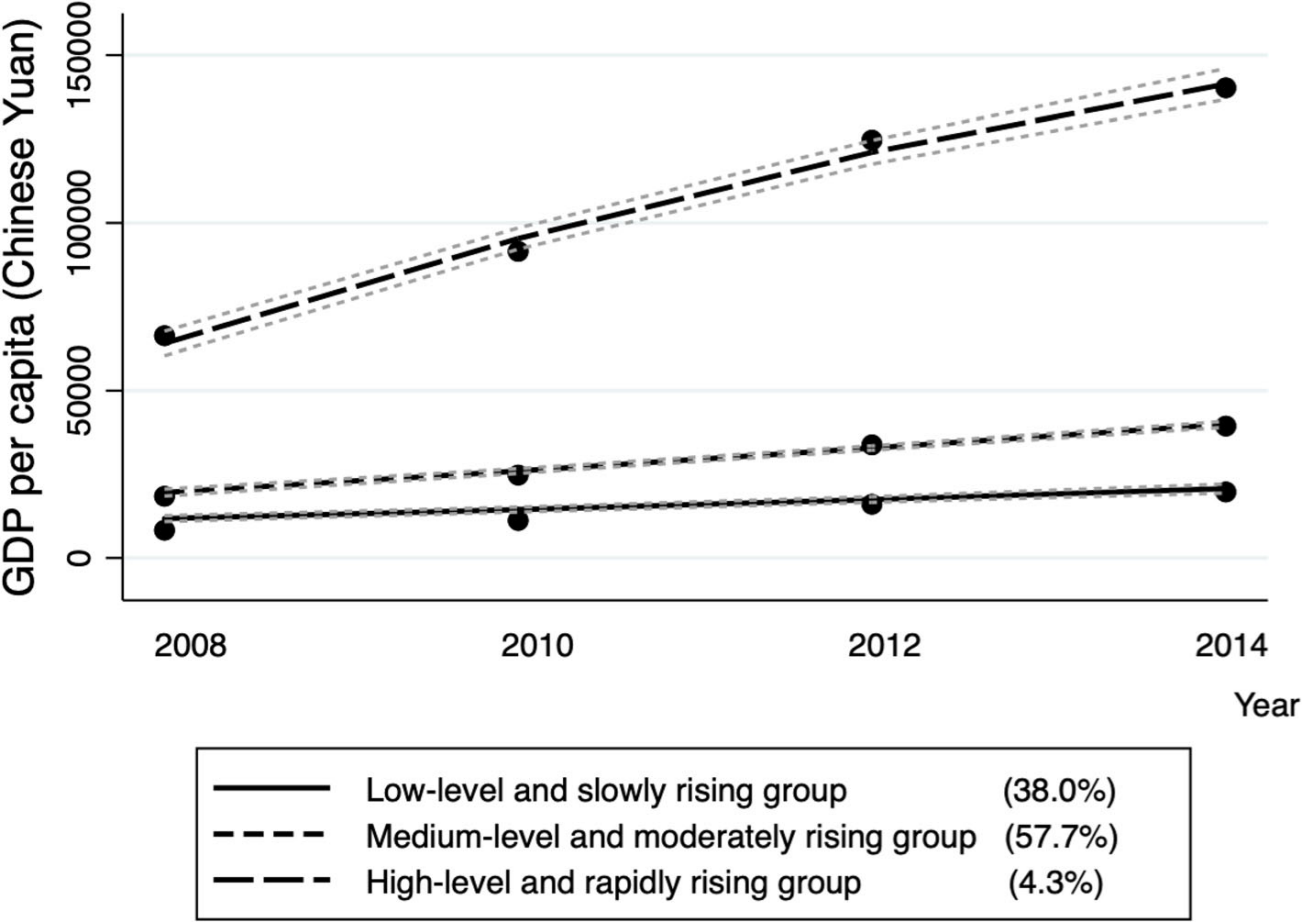
Counties grouped by change in GDP per capita, from group-based trajectory model

The association between the density of health professionals and U5MR might be distinct in various contexts, since economically disadvantaged counties might have fewer health professionals and higher child mortality than economically advantaged ones. Contexts could also capture the types of (unmeasured and often unmeasurable) policies that local businesses and authorities have put into place to accelerate economic growth within counties that may have spillover effects on other factors such as housing availability, water, and sanitation. Thus, we considered the county context in two ways. The first was the county’s poverty status (poor, non-poor) and the second was its change in GDP per capita over time. We grouped all counties by trajectories of their GDP per capita utilizing a group-based trajectory model with a censored normal distribution specification [47, 48]. The group-based trajectory model, a type of finite mixture model, is used here to identify distinct groups of counties following similar developmental trajectories over time [47, 48]. Trajectories are defined by the probability of group membership over time. A three-group model was found to provide the best fit based on the Bayesian information criterion. Group 1, estimated to account for 38.0% of all the counties, was labeled “low level and slowly rising.” Group 2, accounting for 57.7%, was labeled “medium level and moderately rising.” Group 3, accounting for 4.3%, was labeled “high level and rapidly rising” (see Fig. 2).

### Statistical analyses

This study employed a two-way fixed effects model. County-clustered robust standard errors were employed to account for possible auto-correlation and heteroscedasticity [49]. The following is the full model specification.

ln(U5MR_*it*_) = *β*_0_ + *β*_1_*HP*_*it*_ + *β*_2_*HP*_*it*_^∗^*C* + *β*_3_*C* + *γX*_*it*_ + *λ*_*t*_ + *α*_*i*_ + *ε*_*it*_

In the equation, subscript *i* indexes counties and *t* indexes year. U5MR_*it*_ is the under-five mortality rate of county *i* in year *t*. HP is the number of health professionals/1000 population in each county. *C* represents county type defined by poverty status or trajectories of growth in GDP per capita. *X* is a vector of time-varying characteristics of county, including county-level GDP per capita (log), female illiteracy rate, value of equipment per bed (log), and province-level ratio of health expenditure to GDP (dichotomous variable and counties with value larger than or equal to the national average is the reference group). *β*_2_ accounts for the modification effect of county characteristics on the relationship between health professionals and U5MR. *λ* is a fixed effect for discrete years which captures general secular trends. *α* is a time-invariant county-specific fixed effect. *ε*_*it*_ is a county-year-specific random error term. All analyses are conducted using Stata/MP 14.2 (StataCorp, College Station, TX).

### Sensitivity analyses

Multiple sensitivity analyses have been undertaken to check the robustness of findings. We reran the model with the unadjusted U5MR from the county-level Annual Report System on Maternal and Child Health which did not adjust for undercounting of child mortality. We dropped GDP per capita when analyzing the interaction between health professionals and GDP trajectories. Considering that supporting capability to address critical health conditions might affect the relationship between health professionals and U5MR, we analyzed the modification effects of the value of equipment per bed (continuous scale, and as a dichotomous scale using the median value for each year as a cut point) in the relationship between health professionals and U5MR. A clean and appealing built environment could attract more health workers and reduce the possibilities of exposure to risk factors for children, e.g., modern bathroom/toilet in the house. Therefore, we treated sanitation and hygiene infrastructure as confounders using province-level data (proportion of rural population with access to tap water and coverage of sanitary toilets) as proxies for unavailable county-level measures. Finally, because log-transformed health professional density had been used in previous studies, we reran the final model using log-transformed values and compared the difference in results.

## Results

Table 1 shows descriptive statistics for rural Chinese counties every other year over the period 2008–2014. The U5MR dropped by 36.19% in 6 years. Health professionals/1000 population increased from 2.83 to 3.95. Disparities among counties were substantial. Compared to non-poor counties, poor counties had higher U5MR (25.70/1000 live births), lower density of health professionals (2.94/1000 population), and more than twice as large a decrease in U5MR. Counties with low initial level and slowly rising GDP per capita had the highest U5MR (25.81/1000 live births), lowest density of health professionals (2.89/1000 population), and the decrease of U5MR was about twice as large as the other two groups. Counties with high level and rapidly rising GDP per capita had the lowest U5MR (12.96/1000 live births), and the highest density of health professionals (5.81/1000 population).

**Table 1:** Descriptive statistics for rural Chinese counties, 2008–2014

Variables	National						Poverty status					Trajectory of GDP per capita^6^						
	2008	2010	2012	2014			Poor		Non-poor			Low level and slowly rising		Medium level and moderately rising		High level and rapidly rising		
	Mean (SD)	Mean (SD)	Mean (SD)	Mean (SD)	Absolute change^2^	% Change^3^	Mean (SD)	Absolute change^2^	Mean (SD)	Absolute change^2^	Difference^4^	Mean (SD)	Absolute change^2^	Mean (SD)	Absolute change^2^	Mean (SD)	Absolute change^2^	Difference^5^
Under-five mortality rate^1^ (/1000 live births)	23.97 (15.18)	20.50 (12.71)	17.13 (9.52)	15.29 (10.86)	− 8.66	− 36.19	25.70 (16.40)	− 12.68	15.11 (7.03)	− 6.03	10.58	25.81 (16.51)	− 12.72	15.37 (7.08)	− 6.03	12.96 (7.55)	− 7.26	10.44
Total HP in county (/1000 population)	2.83 (1.47)	3.19 (1.52)	3.53 (1.71)	3.95 (1.83)	1.14	39.66	2.94 (1.45)	0.98	3.65 (1.77)	1.24	− 0.71	2.89 (1.39)	0.98	3.51 (1.64)	1.20	5.81 (2.28)	1.75	− 0.62
GDP per capita (log, ¥)	9.46 (0.68)	9.78 (0.67)	10.11 (0.65)	10.28 (0.62)	0.82	8.62	9.43 (0.58)	0.88	10.21 (0.64)	0.78	− 0.78	9.40 (0.54)	0.88	10.13 (0.56)	0.78	11.45 (0.48)	0.79	− 0.73
Female illiteracy rate (%)	15.08 (11.57)	10.68 (9.88)	9.65 (7.77)	10.38 (9.69)	− 4.67	− 31.19	16.34 (13.54)	− 6.02	8.36 (4.90)	− 3.78	7.98	16.45 (13.65)	− 6.07	8.43 (4.93)	− 3.70	8.18 (5.13)	− 4.91	8.02
Provincial ratio of health expenditure to GDP (%)	4.55 (1.36)	5.21 (1.39)	5.46 (1.25)	5.92 (1.28)	1.37	30.08	5.95 (1.40)	1.53	4.86 (1.25)	1.28	1.09	5.95 (1.39)	1.54	4.93 (1.26)	1.28	4.07 (1.04)	1.15	1.01
Value of equipment per bed (log, ¥)	10.24 (0.83)	10.48 (0.66)	10.73 (0.59)	11.01 (0.61)	0.76	7.51	10.38 (0.81)	0.95	10.76 (0.64)	0.64	− 0.39	10.38 (0.81)	0.94	10.73 (0.64)	0.66	11.20 (0.62)	0.62	− 0.35

Numbers in parentheses are standard deviations

*HP* health professionals, *GDP* gross domestic product

^1^Adjusted under-five mortality rate [40]

^2^Difference between year 2008 and 2014, *p* < 0.001 for all variables, from paired *t*-test

^3^Percent of change from 2008 to 2014

^4^Difference between poor counties and non-poor counties, *p* < 0.001, from *t*-test for two independent samples

^5^Difference between counties with low-level and slowly rising GDP per capita and counties with medium-level and moderately rising GDP per capita, *p* < 0.001, from *t* test for two independent samples

^6^One-way analysis of variance for the three groups based on trajectory of GDP per capita shows that not all groups are equivalent (*p* < 0.001). Multiple-comparison tests show that all variables are distinct in each group except the female illiteracy rate between counties with high-level and rapidly rising GDP per capita and counties with medium-level and moderately rising GDP per capita

Table 2 presents the results of the full regression model and interaction analyses. According to model 1 (full model), one additional health professional/1000 population is associated with a 2.6% (*p* < 0.001) reduction in U5MR, after controlling for other covariates. Models 2 and 3 show that county poverty status and GDP trajectories moderate the relationship between health professionals and U5MR. The U5MR reductions attributable to one additional health professional/1000 population are 6.8% (*p* < 0.001) among poor counties, but only 1.1% (*p* = 0.117) among non-poor counties (model 2). These reductions are 6.7% (*p* < 0.001), 0.7% (*p* = 0.326), and 4.3% (*p* = 0.029) in counties with low GDP that slowly increased, medium-level GDP that rose at a moderate pace, and high GDP that rose rapidly, respectively (model 3). Higher GDP per capita, lower female illiteracy rate, higher ratio of provincial health expenditure to GDP, and higher value of equipment per bed are associated with lower U5MR.

**Table 2:** Models for the association between the density of health professionals and the under-five mortality rate in rural China, 2008–2014

Variables	(1)	(2)	(3)
	Full model	Interaction with poverty status	Interaction with GDP trajectory
Total number of health professionals	− 0.026**	− 0.011	− 0.007
	0.007	0.007	0.007
Poverty status (non-poor group as the reference)			
Total HP # poor		− 0.057**	
		0.014	
Trajectory of GDP per capita (medium group as the reference)			
Total HP # slowly rising			− 0.060**
			0.014
Total HP # rapidly rising			− 0.036+
			0.021
GDP per capita (log)	− 0.099**	− 0.080**	− 0.082**
	0.026	0.026	0.026
Female illiteracy rate	0.004*	0.003	0.003
	0.002	0.002	0.002
Provincial health expenditures/GDP (high group as the reference)	0.051**	0.047**	0.048**
	0.015	0.015	0.015
Value of equipment per bed (log)	− 0.017+	− 0.012	− 0.013
	0.009	0.009	0.008
Number of observations	7 653	7 653	7 653
Adjusted *R*^2^ (within group)	0.423	0.427	0.427

*HP* health professionals, *GDP* gross domestic product

Numbers in the cells are fixed effects estimators with their standard errors underneath

+*p* < 0.1; *p < 0.05; **p < 0.01

All models include fixed effects for years and intercepts, but these values are not shown in the table

None of the sensitivity analyses in Table 3 or Additional file 1: Table A2 of Appendix 3 meaningfully change the main conclusions, which suggests that the results presented here are robust. The sensitivity analyses with unadjusted U5MR (model 1), interaction analysis with equipment value per bed (continuous scale in model 3, dichotomous scale in model 4), and the model excluding female illiteracy rate (model 5) do not significantly affect the main conclusions of the relationship between health professionals and U5MR reached with the final model. The results of interaction analysis with trajectories of GDP but without GDP per capita as a covariate (model 2) are similar to those in model 3 of Table 2.

**Table 3:** Sensitivity analyses for the association between the density of health professionals and the under-five mortality rate in rural China, 2008–2014

Variables	(1)	(2)	(3)	(4)	(5)
	Unadjusted U5MR	Interaction with GDP trajectory	Interaction with equipment value (continuous)	Interaction with equipment value (dummy)	Without female illiteracy rate
Total HP	− 0.035**	− 0.007	− 0.042	− 0.024**	− 0.026**
	0.010	0.007	0.044	0.007	0.007
Trajectory of GDP per capita (medium group as the reference)					
Total HP # slowly rising		− 0.065**			
		0.015			
Total HP # rapidly rising		− 0.035+			
		0.021			
Total HP # Value of equipment per bed (continuous)			0.001		
			0.004		
Total HP # Value of equipment per bed (high group as the reference)				− 0.005	
				0.006	
GDP per capita (log)	− 0.087*		− 0.098**	− 0.100**	− 0.103**
	0.041		0.026	0.027	0.026
Female illiteracy rate	0.005*	0.003+	0.004*	0.004*	
	0.002	0.002	0.002	0.002	
Provincial health expenditure/GDP (high group as the reference)	0.037+	0.047**	0.051**	0.051**	0.050**
	0.019	0.015	0.015	0.015	0.015
Value of equipment per bed (log)	− 0.001	−0.014	− 0.021		− 0.018*
	0.012	0.009	0.015		0.008
Value of equipment per bed (high group as the reference)				0.019	
				0.023	
Number of observations	7 618	7 653	7 653	7 653	7 653
Adjusted *R*^2^ (within group)	0.327	0.426	0.423	0.423	0.423

*HP* health professionals, *GDP* gross domestic product

Numbers in the cells are fixed effects estimators with their standard errors underneath

+*p* < 0.1; *p < 0.05; **p < 0.01

All models include fixed effects for years and intercepts, but these values are not shown in the table

Model 1 shows the result when the dependent variable is the log of unadjusted U5MR which does not consider undercounting of child mortality. Model 2 analyzes the modification effects of GDP trajectory on the relationship between health professionals and U5MR, but without GDP per capita as a covariate. Model 3 and model 4 analyze modification effect of equipment value per bed (continuous scale in model 3, dichotomous scale by median of each year in model 4) in the relationship between health professionals and U5MR. Model 5 shows the results without the female illiteracy rate as a covariate

In Additional file 1: Table S2, sensitivity analyses with the proportion of the rural population with access to tap water and coverage of sanitary toilet (models 1–3) lower the magnitude of the association between health professionals and U5MR, but do not otherwise affect the main conclusions of the relationship from those of the main model. Models 4–6 using the log-transformed density of health professionals as predictors show the same direction as the main model.

## Discussion

This study found that recent increases in health professionals were associated with reductions in child mortality in rural Chinese counties. Further, the magnitude of this effect depends on local economic development status; the greatest effects were seen among poor counties and counties with low initial and slowly rising GDP per capita. Increasing health professionals among poor counties could decrease U5MR by 5.7% more (*p* < 0.001) than a similar investment within non-poor counties. Similarly, investing human resources for health in counties with low initial level and slowly rising GDP could decrease U5MR by 6.0% more (*p* < 0.001) than doing so in counties with medium initial level and moderately rising GDP. A set of sensitivity analyses support the robustness of these results.

Our findings are consistent with and update previous Chinese county-level studies, such as those by Anand et al. and Guo et al. [16, 29]. Our study is also congruent with the international literature using local (county) level data. A one-unit increase in health professionals (excluding pediatricians) was associated with a 47% reduction in U5MR in 366 “Secondary Tier of Medical Care Units” in Japan [21]; a one-unit increase in physician density was associated with 2.3% reduction in neonatal mortality in 4267 counties (municipalities) in Brazil [22]. However, these findings differ from some province-level studies in China. Feng et al. reported that the “health system and policy” factor (that included density of health workers) was positively associated with U5MR, but this analysis also controlled for health care services which are likely to be mediators of this relationship [2]. Sun illustrated that physician density was positively, but statistically insignificantly, associated with IMR [9]. It is important to note that studies using province-level data are unable to reflect variations within a province. The high levels of county-level heterogeneity within provinces may help explain these seemingly contradictory findings. For instance, using 2014 data, in Anhui Province, county GDP per capita differed by as much as 12.39 times between the richest and poorest county and this corresponded to a 2.35 times difference in the number of health professionals/1000 population.

The relationship between health professionals and child mortality has been well established, but the magnitude varies by local economic context. The magnitude is much larger among poor counties; one more health professional/1000 population could reduce U5MR by 6.8% in poor counties but 1.1% in non-poor counties. Results from previous study corroborate our findings. It was found that in poor rural counties, the number of health professionals per capita was negatively associated with IMR [29]. Counties officially designated as poor qualify for additional support, such as financial subsidies and tax deductions, from both the Central and province-level governments [50]. However, these counties are still less attractive for health professionals due to their low economic and social development level and likely living conditions. Nevertheless, increasing the density of health professionals in these counties would contribute to a greater reduction in U5MR than placing these professionals in other locations.

The relationship between health professionals and U5MR among rural counties with different GDP trajectories exhibits different patterns: it was the strongest among counties with low initial level and slowly rising of GDP per capita, followed by those with high level and rapidly rising GDP. Rapid and sustained economic growth is likely to bring a host of improvements in many determinants of U5MR, while those counties that did not experience such growth will remain dependent on health workers as the prime driver of child mortality reductions, all else equal. Moreover, with sustained low levels of economic support, it is hard for these counties to attract and retain health workers and to provide enough health care services [51]. However, the counties with high-level and rapidly rising GDP contain the most health professionals/1000 population, have the lowest U5MR, and have also seen the most substantial drop in U5MR (reduction of 7.26/1000 live births in 6 years). Subgroup analysis (not shown) suggests that the elasticity of U5MR to GDP per capita was strongest in these counties (*b* = − 0.156, *p* = 0.064). Moreover, this analysis considering different GDP trajectories is consistent with theories of economic growth that emphasize that such growth is not only a product of basic endowments, but must also incorporate changes in technology and accumulation of capital and other investments over time [52]. Such growth is considered by most economists to be endogenous in that it depends on different choices made often early in the observed time period [52]. It seems therefore logical that such differences could have wide-ranging effects on health and health care, as our empirical results suggest.

China’s achievements in reducing child mortality were mainly attributable to supportive curative services and, more importantly, prevention (primary, secondary, and tertiary) services [53, 54]. Primary prevention services include health education, pre-pregnancy medical examination, folic acid supplementation, and pregnancy health care [55]. Secondary preventive measures include provision of available and appropriate treatment of diseases in township centers, and prenatal diagnosis in provincial/municipal/county hospitals which allows for necessary pregnancy termination [19, 56]. Tertiary preventive measures include early curative services such as early surgical treatment which could directly improve the survival rate of children [19]. Increases in the health care workforce contributed to the supply and effective implementation of these services. However, insufficient health care workforce could limit the provision of these services and increase child mortality rates. For example, a study in Guizhou province and Shaanxi province demonstrated that 30% of deaths among 0–59-month-old children in 2008 could be prevented by 2015 if primary health care intervention coverage had expanded to a more feasible level [15].

The Chinese health reform since 2009 proposed several measures to support health systems in economically disadvantaged areas. For instance, developed areas helped their counterparts in poverty-stricken areas to develop a health system with long-term and stable counterpart support mechanism; Central and provincial governments increased the level of transfer payments to economically disadvantaged areas; health professionals were incentivized to work in central and western China; the construction of health facilities in remote areas was also accelerated [33, 35]. Although the government realized the importance of investment in economically disadvantaged areas, there is still a long way to go.

This study has several strengths. First, this is the first ever longitudinal study evaluating the association between the density of health professionals and U5MR at the county level in rural China with nationally representative datasets. Second, time-invariant unobserved or unobservable county characteristics are controlled by county fixed effects, such as unmeasured geographical and sociocultural characteristics [49]. The within R-square of the full model indicates that it explains up to 42.3% of the within-county variation in U5MR from 2008 to 2014. Besides, the fixed effects for years additionally control the secular trends and technical progress to some extent [17]. Third, most existing studies exploring the heterogeneity of local contexts have focused primarily on time-invariant characteristics, but this study identified a new and important dynamic factor that may affect U5MR: the local level and pace of economic growth.

Study limitations are as follows. The foremost limitation of any ecological study is the possibility of ecological fallacy. Specifically, it is impossible to determine with certainty whether the child who would have died was the same who benefited from increased availability of health professionals, since child-level data are not available. Meanwhile, the mere availability of health professionals may not reflect the accessibility of children to life-saving health care services [57]. Nonetheless, accumulating evidence has confirmed the importance of health professionals in improving the determinants of the child survival rate [2, 3, 17–19]. In addition, the fixed effect specification means that the results are “conditional” on year and counties involved in this study, so one should be cautious in making inferences to other years or places [49]. This study may suffer from several time-variant omitted variables, such as the capability of health professionals [14], as an adequately sized, committed, motivated health professional with public health and clinical competencies is a prerequisite for reducing U5MR [17]. Some omitted variables could be proxied by factors included in the model, such as GDP per capita which has been argued to capture several confounding factors (e.g., nutrition, access to clean water) [14]. Although we have used panel data with fixed effects specifications and performed several sensitivity analyses, there are still possible sources of biases that we could not account for and this study should therefore be considered to be associational rather than causal in nature.

The dataset also has some limitations. Health professionals in this study include all specialties not merely pediatricians, but outcomes are only measured among children, which may bias our estimates towards the null. The dataset only considers the number of health professionals, but it should be noted that health worker quality and capacity (which is often reflected by education level) may differ by year and region. The proportion of licensed physicians (including licensed assistant physicians) with a bachelor degree or above was 47.5% for all health institutions, and 11.9% for township health centers in 2014, an increase of 5.1 and 3.1 percentage points compared with 2009, while the increase in the proportion of registered nurses was 5.0 and 2.2 percentage points [36]. The effects of improved quality over the years were absorbed by the increased number of health professionals, which might overestimate their influence. Additionally, although mortality undercounting has been improved in recent years, it is widely recognized that vital statistics registries do not fully capture all child mortality, so the main model using the adjusted U5MR should be considered more reliable [40].

## Conclusions

This study finds that increased health professionals are associated with reduced U5MR, underlining the importance of investing in the health care workforce to further strengthen rural Chinese health systems. Meanwhile, the stronger effects seen for health professionals in poor counties and those with low initial level and slowly rising GDP per capita justify further investments in these areas, such as recruiting new health professionals or reorganizing health professionals between different counties. Finally, the results of this study could be instructive for other developing countries striving to achieve SDG3 by identifying county-level characteristics likely to indicate where allocating additional health professionals may have the greatest influence.

## Additional file

Additional file 1: **Appendix 1: Table A1:** Information on missing data. **Appendix 2:** Imputation approach for female illiteracy rate. **Appendix 3: Table A2:** Additional sensitivity analyses for the association between the density of health professionals andthe under-five mortality rate in rural China, 2008-2014. (DOCX 37 kb)

## Abbreviations

GDP: Gross domestic product
HP: Health professional
IMR: Infant mortality rate
U5MR: Under-five mortality rate

## Footnotes

1 Formal medical training for physicians has three levels in China: medical college (5 years of medical education or more after 12 years of primary and secondary education to get a bachelor’s degree of medicine or above), junior medical college (3 years of medical education after 12 years of primary and secondary education), and technical school (3 years of medical education after 9 years of primary and secondary education) [51]. Completion of medical college is required to become a licensed physician, and junior medical college training is required to become a licensed assistant physician, both of which also need to pass the National Practicing Physician (or Assistant Physician) Examination and periodic government assessments [51]. Due to this requirement, licensed assistant physicians have to work for several years before applying for their physician license. Furthermore, once they are licensed, assistant physicians assume nearly the same duties as licensed physicians, especially in primary health care institutions. There is not any other assistant role defined in official Chinese health professional categorization. For this reason, we only include assistant physicians.

## References

[1] United Nations. Goal 3: Ensure healthy lives and promote well-being for all at all ages. https://www.un.org/sustainabledevelopment/health/. Accessed 18 Aug 2018.

[2] Feng XL, Theodoratou E, Liu L, Chan KY, Hipgrave D, Scherpbier R (2012). Social, economic, political and health system and program determinants of child mortality reduction in China between 1990 and 2006: a systematic analysis. J Glob Health.

[3] Mosley W, Chen L (1984). An analytical framework for the study of child survival in developing countries. Popul Develop Rev.

[4] Keats EC, Macharia W, Singh NS, Akseer N, Ravishankar N, Ngugi AK (2018). Accelerating Kenya’s progress to 2030: understanding the determinants of under-five mortality from 1990 to 2015. BMJ Glob Health.

[5] Corsi DJ, Subramanian SV (2014). Association between coverage of maternal and child health interventions, and under-5 mortality: a repeated cross-sectional analysis of 35 sub-Saharan African countries. Glob Health Action.

[6] Acheampong M, Ejiofor C, Salinas-Miranda A (2017). An analysis of determinants of under-5 mortality across countries: defining priorities to achieve targets in Sustainable Developmental Goals. Matern Child Health J.

[7] Bishai DM, Cohen R, Alfonso YN, Adam T, Kuruvilla S, Schweitzer J (2016). Factors contributing to maternal and child mortality reductions in 146 low- and middle-income countries between 1990 and 2010. PLoS One.

[8] Yalcin SS, Tezel B, Kose MR, Tugay D, Mollahaliloglu S, Erkoc Y (2013). Changes and determinants in under-five mortality rate in Turkey since 1988. Cent Eur J Public Health.

[9] Sun J. Health performance and regional differences of government health expenditure in China: empirical analysis based on provincial panel data. Wuhan University Journal (Philosophy & Social Science). 2011;64(06):75–80 (in Chinese).

[10] Luo S, Wang Y, Gao J, Xu L (2006). Study on infant mortality rate and related factors in China in 1998-2003. Mater Child Health Care China.

[11] Anand S, Barnighausen T (2004). Human resources and health outcomes: cross-country econometric study. Lancet..

[12] Speybroeck N, Kinfu Y, Poz MRD, Evans DB (2006). Reassessing the relationship between human resources for health, intervention coverage and health outcomes.

[13] Anand S, Bärnighausen T (2012). Health workers at the core of the health system: framework and research issues. Health Policy.

[14] Nguyen MP, Mirzoev T, Le TM (2016). Contribution of health workforce to health outcomes: empirical evidence from Vietnam. Hum Resour Health.

[15] Jiang Z, Guo SF, Scherpbier RW, Wen CM, Xu XC, Guo Y (2015). Determining optimal strategies to reduce maternal and child mortality in rural areas in western China: an assessment using the lives saved tool. Biomed Environ Sci.

[16] Anand S, Fan VY, Zhang J, Zhang L, Ke Y, Dong Z (2008). China’s human resources for health: quantity, quality, and distribution. Lancet..

[17] Farahani M, Subramanian SV, Canning D (2009). The effect of changes in health sector resources on infant mortality in the short-run and the long-run: a longitudinal econometric analysis. Soc Sci Med.

[18] Muldoon KA, Galway LP, Nakajima M, Kanters S, Hogg RS, Bendavid E (2011). Health system determinants of infant, child and maternal mortality: a cross-sectional study of UN member countries. Glob Health.

[19] Cui H, He C, Kang L, Li Q, Miao L, Shen L (2016). Under-5-years child mortality due to congenital anomalies: a retrospective study in urban and rural China in 1996-2013. Am J Prev Med.

[20] Shi L, Macinko J, Starfield B, Xu J, Regan J, Politzer R (2004). Primary care, infant mortality, and low birth weight in the states of the USA. J Epidemiol Commun H.

[21] Sakai R, Fink G, Kumamaru H, Kawachi I (2016). The impact of pediatrician supply on child health outcomes: longitudinal evidence from Japan. Health Serv Res.

[22] Sousa A, Dal Poz MR, Boschi-Pinto C (2013). Reducing inequities in neonatal mortality through adequate supply of health workers: evidence from newborn health in Brazil. PLoS One.

[23] Fernandes QF, Wagenaar BH, Anselmi L, Pfeiffer J, Gloyd S, Sherr K (2014). Effects of health-system strengthening on under-5, infant, and neonatal mortality: 11-year provincial-level time-series analyses in Mozambique. Lancet Glob Health.

[24] Akinkugbe O, Mohanoe M (2009). Public health expenditure as a determinant of health status in Lesotho. Soc Work Public Health.

[25] Anyanwu JC, Erhijakpor AE (2009). Health expenditures and health outcomes in Africa. Afr Dev Rev.

[26] Morley CP, Wang D, Mader EM, Plante KP, Kingston LN, Rabiei A (2017). Analysis of the association between millennium development goals 4 & 5 and the physician workforce across international economic strata. BMC Int Health Hum Rights.

[27] Carr-Hill R, Currie E (2013). What explains the distribution of doctors and nurses in different countries, and does it matter for health outcomes?. J Adv Nurs.

[28] Chen Y. A study on the factors associated with infant mortality rates in China. Chengdu: Sichuan University; 2006. (in Chinese).

[29] Guo S, Wang L, Zhang W (2001). Cross-county analysis on infant mortality in remote and poverty-stricken areas in China. China Public Health.

[30] Zhang Z (2015). Research on health performance of government health expenditure based on provincial panel data. Stat Decis.

[31] Or Z, Wang J, Jamison D (2005). International differences in the impact of doctors on health: a multilevel analysis of OECD countries. J Health Econ.

[32] World Bank, World Health Organization, Ministry of Finance, P.R. China, National Health and Family Planning Commission, P.R. China, Ministry of Human Resources and Social Security, P.R. China. Healthy China: Deepening health reform in China, building high-quality and value-based service delivery. 2016. http://www.wpro.who.int/china/publications/health-reform-in-china.pdf. Accessed 10 Nov 2018.

[33] CPC Central Committee. Opinions of the CPC Central Committee and the State Council on deepening the health care system reform. 2009. http://www.gov.cn/jrzg/2009-04/06/content_1278721.htm. Accessed 10 Nov 2018. (in Chinese).

[34] Liu Q, Wang B, Kong Y, Cheng KK (2011). China’s primary health-care reform. Lancet..

[35] State Council (2009). Notice of the state council on issuing the plan on recent priorities in carrying out the reform of health care system (2009–2011).

[36] National Health and Family Planning Commission of the People’s Republic of China (2015). 2015 China Statistics Yearbook.

[37] The State Council Leading Group Office of Poverty Alleviation and Development. The list of National poverty alleviation and development focus counties. 2012. http://www.cpad.gov.cn/art/2012/3/19/art_343_42.html. Accessed 30 Jan 2019. (in Chinese).

[38] The identification of National poverty alleviation and development focus counties and counties in contiguous poor areas. 2013. http://www.gov.cn/gzdt/2013-03/01/content_2343058.htm. Accessed 30 Jan 2019. (in Chinese).

[39] The establishment of retreat mechanism for National poverty alleviation and development focus counties. 2008. http://nys.mof.gov.cn/zhengfuxinxi/bgtDiaoCheYanJiu_1_1_1_1_2/200807/t20080717_57786.html. Accessed 30 Jan 2019. (in Chinese).

[40] Wang YP, Li XH, Zhou MG, Luo SS, Liang J, Liddell CA (2016). Under-5 mortality in 2851 Chinese counties, 1996-2012: a subnational assessment of achieving MDG 4 goals in China. Lancet..

[41] Liu L, Oza S, Hogan D, Chu Y, Perin J, Zhu J (2016). Global, regional, and national causes of under-5 mortality in 2000-15: an updated systematic analysis with implications for the Sustainable Development Goals. Lancet..

[42] McGuire JW (2006). Basic health care provision and under-5 mortality: a cross-national study of developing countries. World Dev.

[43] Marmot M, Friel S, Bell R, Houweling TA, Taylor S (2008). Closing the gap in a generation: health equity through action on the social determinants of health. Lancet..

[44] Kominski GF (2014). Changing the U.S. health care system : key issues in health services policy and management. 4th ed.

[45] Gakidou E, Cowling K, Lozano R, Murray CJL (2010). Increased educational attainment and its effect on child mortality in 175 countries between 1970 and 2009: a systematic analysis. Lancet..

[46] Guanais FC (2013). Municipal-level covariates of health status in Brazil: a proposed method for data interpolation. Rev Panam Salud Publica.

[47] Jones BL, Nagin DS (2013). A note on a stata plugin for estimating group-based trajectory models. Sociol Methods Res.

[48] Nagin DS, Odgers CL (2010). Group-based trajectory modeling in clinical research. Annu Rev Clin Psychol.

[49] Wooldridge J (2014). Introductory econometrics: a modern approach.

[50] Ministry of Finance, Poverty Alleviation Office, National Development and Reform Commission, National Civil Affairs Commission, Ministry of Agriculture, Forestry Bureau. Issuing the measures for the administration of special government finance funds for poverty alleviation. 2011. http://www.mof.gov.cn/zhengwuxinxi/caizhengwengao/2012wg/wg201201/201203/P020120331502249426625.doc. Accessed 13 Dec 2018. (in Chinese).

[51] Li X, Lu J, Hu S, Cheng KK, Maeseneer JD, Meng Q (2017). The primary health-care system in China. Lancet..

[52] Salvadori N (2003). Old and new growth theories: an assessment.

[53] Rudan I, Chan KY, Zhang JS, Theodoratou E, Feng XL, Salomon JA (2010). Causes of deaths in children younger than 5 years in China in 2008. Lancet..

[54] Liu YL, Rao KQ, Wu J, Gakidou E (2008). China’s health system performance. Lancet..

[55] Ministry of Health of the People’s Republic of China. Report on prevention and treatment of birth defects in China (2012). 2012. http://www.gov.cn/gzdt/2012-09/12/content_2223371.htm. Accessed 6 Mar 2019. (in Chinese).

[56] Liu S, Joseph KS, Kramer MS, Allen AC, Sauve R, Rusen ID (2002). Relationship of prenatal diagnosis and pregnancy termination to overall infant mortality in Canada. JAMA..

[57] Starfield B, Shi L, Macinko J (2005). Contribution of primary care to health systems and health. Milbank Q.

