# Supplementary material for: The impact of the health care workforce on under-five mortality in rural China
Source: Hum Resour Health. 2019 Mar 18;17:21. doi: 10.1186/s12960-019-0357-5 (PMC6423838; doi:10.1186/s12960-019-0357-5)
Supplement: Supplementary file 1 — Appendix 1: Table A1: Information on missing data. Appendix 2: Imputation approach for female illiteracy rate. Appendix 3: Table A2: Additional sensitivity analyses for the association between the density of health professionals andthe under-five mortality rate in rural China, 2008-2014. (DOCX 37 kb) [file 12960_2019_357_MOESM1_ESM.docx]

**Additional file**

**Appendix 1**

**Table A1: Information on missing data**

| Variables | Missing | Total | Percent Missing |
| --- | --- | --- | --- |
| Under-five mortality rate | 42 | 7,956 | 0.53 |
| GDP per capita | 185 | 7,956 | 2.33 |
| Female illiteracy rate^1^ | 71 | 7,956 | 0.89 |
| Trajectory of GDP per capita | 233 | 7,956 | 2.93 |
| Value of equipment per bed | 22 | 7,956 | 0.28 |
| Total HP in county | 0 | 7,956 | 0 |
| Provincial ratio of health expenditure to GDP | 0 | 7,956 | 0 |

* GDP: Gross Domestic Product; HP: Health Professionals

* 1. Before imputation, 75.21% (5984/7956) of the observations were missing for female illiteracy rate. After imputation, there were 71 observations with missing values which correspond to the counties that were created between 2000 and 2010.

**Appendix 2: Imputation approach for female illiteracy rate**

We utilize a novel approach validated previously for obtaining consistent estimates of missing data within the context of a federal country where municipal (county) data are routinely aggregated to state and regional levels. This approach was developed for estimating determinants of local economic growth in the United States and more recently adapted for use in Brazil--another large federal country [46]. We use this approach to impute data for the female illiteracy rate.

The only available data of female illiteracy rate at the county level is in year 2010 (census year). Based on the previously mentioned approach, we impute the female illiteracy rate in 2008, 2012 and 2014 using non-linear interpolation and extrapolation methods that model within-county changes in relation to existing values at the county level and contemporaneous values at the province level. This approach combines county-level data available for census years with province-level data available for every year.

The interpolation of county-level data between two census years (2000 and 2010) follows a three-step method beginning with Equation 1:

$$X_{c,2000+t}=X_{c,2000+t-1}*e^{\left( \left( \frac{\ln\left( \frac{X_{p,2000+t}}{X_{p,2000+t-1}} \right)}{\ln\left( \frac{X_{p,2010}}{X_{p,2000}} \right)} \right)*\ln\left( \frac{X_{c,2010}}{X_{c,2000}} \right) \right)} Equation 1$$

In Equation 1, subscript c indexes counties; p indexes provinces; t indexes inter-census years ranges from 1 to 9; 2000 is the year of the first census survey; 2010 is the year of the second census survey. X is the variable to be imputed (female illiteracy rate). The values of X_c,2000_, X_c,2010_, and the set of 11 elements from X_p,2000_ to X_p,2010_ are known as priori.

To avoid the possibility that imputed data may grow exponentially and turn out to be implausibly high or low, Equation 2 is used to smoothen the deviation from the linear interpolation.

$$\Upsilon_{c,2000+t}=\frac{1}{10}\left( X_{c,2010}-X_{c,2000} \right)+\Upsilon_{c,2000+t-1} Equation 2$$

In Equation 2, γ is the same meaning as X. The values of X_c,2000_ and X_c,2010_ are known as priori, and γ_c,2000_=X_c,2000_.

To limit possible deviation from the linear interpolation, Equation 3 is specified as follows.

$$Z_{c,2000+t}=\Upsilon_{c,2000+t}+\frac{X_{c,2000+t}-\Upsilon_{c,2000+t}}{e^{(0.03*\left| X_{c,2000+t}-\Upsilon_{c,2000+t} \right|)}} Equation 3$$

In Equation 3, Z is the same meaning as X.

County-level data for 2000 and 2010 were obtained from National population census database, and province-level data from 2000 to 2014 were obtained from the China Health and Family Planning Statistical Yearbook.

**Appendix 3**

**Table A2: Additional sensitivity analyses for the association between the density of health professionals and the under-five mortality rate in rural China, 2008-2014**

| Variables | (1) | (2) | (3) | (4) | (5) | (6) |
| --- | --- | --- | --- | --- | --- | --- |
|  | Add sanitation covariates | | | Use total HP (log) as predictor | | |
| Total number of HP | -0.016* | -0.001 | 0.001 |  |  |  |
|  | 0.007 | 0.007 | 0.007 |  |  |  |
| Total number of HP (log) |  |  |  | -0.074* | -0.008 | -0.0002 |
|  |  |  |  | 0.029 | 0.032 | 0.032 |
| Poverty status  (non-poor group as the reference) |  |  |  |  |  |  |
| Total HP # poor |  | -0.059** |  |  |  |  |
|  |  | 0.014 |  |  |  |  |
| Total HP # poor (log) |  |  |  |  | -0.159** |  |
|  |  |  |  |  | 0.044 |  |
| Trajectory of GDP per capita  (medium group as the reference) |  |  |  |  |  |  |
| Total HP # slowly rising |  |  | -0.058** |  |  |  |
|  |  |  | 0.014 |  |  |  |
| Total HP # rapidly rising |  |  | -0.030 |  |  |  |
|  |  |  | 0.021 |  |  |  |
| Total HP # slowly rising (log) |  |  |  |  |  | -0.159** |
|  |  |  |  |  |  | 0.045 |
| Total HP # rapidly rising (log) |  |  |  |  |  | -0.236* |
|  |  |  |  |  |  | 0.105 |
| GDP per capita (log) | -0.101** | -0.081** | -0.083** | -0.094** | -0.076** | -0.079** |
|  | 0.027 | 0.026 | 0.026 | 0.026 | 0.026 | 0.026 |
| Female illiteracy rate | 0.005** | 0.004* | 0.004* | 0.003* | 0.002 | 0.002 |
|  | 0.002 | 0.002 | 0.002 | 0.002 | 0.002 | 0.002 |
| Provincial health expenditures/GDP  (high group as the reference) | 0.023 | 0.017 | 0.018 | 0.046** | 0.041** | 0.042** |
|  | 0.015 | 0.015 | 0.015 | 0.015 | 0.015 | 0.015 |
| Value of equipment per bed (log) | -0.021* | -0.017* | -0.018* | -0.017+ | -0.013 | -0.014+ |
|  | 0.009 | 0.009 | 0.009 | 0.009 | 0.008 | 0.008 |
| Proportion of rural population  with access to tap water | 0.009** | 0.009** | 0.009** |  |  |  |
|  | 0.001 | 0.001 | 0.001 |  |  |  |
| Coverage of sanitary toilet | -0.003** | -0.003** | -0.003** |  |  |  |
|  | 0.001 | 0.001 | 0.001 |  |  |  |
| Number of observations | 7482 | 7482 | 7482 | 7653 | 7653 | 7653 |
| Adjusted R^2^ (within group) | 0.443 | 0.447 | 0.447 | 0.422 | 0.425 | 0.425 |

* HP: Health Professionals; GDP: Gross Domestic Product

* Numbers in the cells are fixed effects estimators with their standard errors underneath

* +p<0.1; *p<0.05; **p<0.01

* All models include fixed effects for years and intercepts, but these values are not shown in the table

* Data on the proportion of the rural population with access to tap water and coverage of sanitary toilets are not available for the 171 observations in Tibet Province

* Models 1-3 show results when the proportion of the rural population with access to tap water and coverage of sanitary toilets are added. Models 4-6 show the results when the predictor is the log of total number of health professionals per 1,000 population
